# A Total Bounded Variation Approach to Low Visibility Estimation on Expressways

**DOI:** 10.3390/s18020392

**Published:** 2018-01-29

**Authors:** Xiaogang Cheng, Bin Yang, Guoqing Liu, Thomas Olofsson, Haibo Li

**Affiliations:** 1 College of Telecommunications and Information Engineering, Nanjing University of Posts and Telecommunications, Nanjing 210003, China; haiboli@kth.se; 2 Department of Applied Physics and Electronics, Umeå University, 90187 Umeå, Sweden; bin.yang@umu.se (B.Y.); thomas.olofsson@umu.se (T.O.); 3 School of Electrical Engineering and Computer Science, Royal Institute of Technology, 10044 Stockholm, Sweden; 4 School of Environmental and Municipal Engineering, Xi’an University of Architecture and Technology, Xi’an 710055, China; 5 School of Physical and Mathematical Sciences, Nanjing Tech University, Nanjing 211800, China; guoqing@njtech.edu.cn

**Keywords:** total bounded variation, image spectrum, low visibility estimation, piece stationary, fog and haze

## Abstract

Low visibility on expressways caused by heavy fog and haze is a main reason for traffic accidents. Real-time estimation of atmospheric visibility is an effective way to reduce traffic accident rates. With the development of computer technology, estimating atmospheric visibility via computer vision becomes a research focus. However, the estimation accuracy should be enhanced since fog and haze are complex and time-varying. In this paper, a total bounded variation (TBV) approach to estimate low visibility (less than 300 m) is introduced. Surveillance images of fog and haze are processed as blurred images (pseudo-blurred images), while the surveillance images at selected road points on sunny days are handled as clear images, when considering fog and haze as noise superimposed on the clear images. By combining image spectrum and TBV, the features of foggy and hazy images can be extracted. The extraction results are compared with features of images on sunny days. Firstly, the low visibility surveillance images can be filtered out according to spectrum features of foggy and hazy images. For foggy and hazy images with visibility less than 300 m, the high-frequency coefficient ratio of Fourier (discrete cosine) transform is less than 20%, while the low-frequency coefficient ratio is between 100% and 120%. Secondly, the relationship between TBV and real visibility is established based on machine learning and piecewise stationary time series analysis. The established piecewise function can be used for visibility estimation. Finally, the visibility estimation approach proposed is validated based on real surveillance video data. The validation results are compared with the results of image contrast model. Besides, the big video data are collected from the Tongqi expressway, Jiangsu, China. A total of 1,782,000 frames were used and the relative errors of the approach proposed are less than 10%.

## 1. Introduction

Low visibility caused by heavy fog and haze, especially the dumpling fog in the waterfront area, remains a great threat to expressway traffic safety. For instance, on 6 November 2016 [1] in Pudong, Shanghai, China, nine people were killed and over 40 people were injured in two traffic accidents resulting from heavy fog. Besides, 144 traffic accidents occurred in Dubai on 12 January 2017 because of heavy fog [2]. Real-time perception and human intervention are both vital methods to cut down traffic accidents in virtue of fog and haze. Nowadays, there are two types of visibility estimation methods: optics based method and vision based method. The main drawbacks of the optics based method are the limited spatial volume of sampling and the high cost in the implementation [3]. The advantages of vision based method are low costs and easiness when it came to the accomplishment of the visibility map of the road network, the method turned out to be a research focus in recent years. However, the estimation accuracy should be improved because of the complexity and time-varying characteristics.

Koschmieder [4] presented an atmospheric visibility formula, in which an exponential relation model between various variables, e.g., luminance, observed visibility and extinction coefficient of aerosols, was established. The Koschmieder formula (Koschmieder law) laid the foundation for the atmospheric visibility estimation. Blackwell [5] explored observed threshold values of human eyes on account of a subjective observation method later. Based on [4,5], different kind of vision-based methods of visibility estimation are presented, such as the exploratory studies in the early stages [6,7,8], the luminance curves models [9,10,11,12], image contrast models [13,14,15,16,17], road sign models [18,19,20,21,22,23], regression models [24,25], etc. However, regarding practical applications, the current vision based methods are facing some challenges: (1) How should different visibility situations be handled? The situations include different visibility value intervals and different kinds of road. One method cannot be used to estimate all visibility. It is a reasonable choice to design different algorithms according to different situations. The situations with more traffic accidents should be firstly focused on. (2) The methods above were not verified by big data collected from real world. As such, big estimation errors could happen in practical application. This study aims to overcome the above drawbacks. Therefore, a novel low-visibility estimation approach based on image spectrum and TBV, hereinafter referred to as S-TBV, is presented. The contributions of this paper can be presented as follows:

(1) It is the very first time that image spectrum and TBV were applied to characterize features of fog and haze and estimate visibility. From the practical standpoint, the expressway visibility of less than 300 m caused by heavy fog and haze, being more dangerous, was chiefly explored. While the visibility and the high frequency (HF) coefficient ratios of image Fourier transform (discrete cosine) were increasing, low frequency (LF) coefficient ratios decrease correspondingly. The TBV of foggy and hazy images climbed. For foggy and hazy images with visibility of less than 300 m, HF coefficient ratios were under 20%, and LF coefficient ratios ranged from 100% to 120%. Based on this spectrum feature, foggy and hazy images with low visibility images can be sorted out, and the TBV trend was consistent with the trend of foggy and hazy visibility.
(2) Considering the polynomial regression and piecewise stationary time series analysis, a nonlinear relationship between TBV and real visibility was established.
(3) To overcome the effect of different road landscape and sunshine luminance, the relative ratio of image spectrum and total bounded variation were adopted.
(4) Unlike the current visibility estimation methods (model-driven), the method proposed in this study is a semi-data-driven approach. It is the very first time that a big dataset (1,782,000 frames) collected from real world, Tongqi expressway, China, was used to train the semi-data-driven model. The proposed approach was validated by the big video data.

The structure of this paper is listed as follows. In Section 2, the related works are introduced. In Section 3, the definition of visibility is introduced and the application of the algorithm is then elaborated. Furthermore, the TBV approach is introduced in detail. Firstly, the image spectral feature of foggy and hazy images is discussed. Secondly, the rationality of TBV in characterizing the feature of foggy and hazy images is analyzed, and an innovative piecewise and stationary function is established. In Section 4 and Section 5, validated results for the algorithm are analyzed based on surveillance videos from Chinese expressways. Finally, conclusions are made.

## 2. Related Works

Based on the Koschmieder law [4] and the human eye threshold [5], some exploratory studies of atmospheric visibility estimation were presented in the early stages. Middleton and Mungall [6] assumed that the contrast threshold value of eyes was 0.02. An inverse proportional relationship between visibility and extinction coefficient was established as *Vis* = 3.9/*k*, in which the *Vis* is the atmospheric visibility and *k* is the extinction coefficient. Horvath [7] verified the feasibility of estimating the atmospheric visibility based on Koschmieder’s formula, and analyzed possible errors. According to [4], Steffens [8] estimated the atmospheric visibility with black and white photo, which is considered to be a pioneering exploration of the vision based method. Nevertheless, there was no breakthrough in the vision based method for a few decades due to limitations in imaging technologies. Fortunately, Bell laboratories verified the possibility of producing a charge-coupled device (CCD) in 1969. After that, Fairchild Semiconductor developed the CCD image sensors in 1973. In the 20 years following 1973, the development of semiconductor and computer technologies was booming, which laid good hardware foundations for academic research of the vision based method.

Recently, the luminance curve models of vision based method are rapidly developed. In these models, the luminance curves are often used independently, or combined with other parameters [9,10,11,12]. Hautière, et al. [9] proposed an applicable visibility estimation method, and he, based on Koschmieder’s law, put forward the rigorous mathematical derivation. One type of the luminance curve was firstly proposed and their inflection points were collected by second derivatives of luminance curves. The method proposed in [9] had the probability of practical application for the first time, and it laid the foundation for the video based subjective visibility observation. Based on [9], Lenor, et al. [10], Negru and Nedevschi [11] studied visibility estimations further with luminance curves. Lenor [10] introduced a model with the theory of radiative transfer. Through modeling in-scattered light, a relationship between extinction coefficients of atmosphere and inflection points of luminance curves can be established. In [11], the presence of fog was perceived based on the fog’s density estimation. When the horizon line and the inflection point in fog images were acquired, the fog visibility can therefore be calculated. Guo, et al. [12] presented a visibility estimation method based on the combination of camera parameter estimation and region of interest (ROI) search. The position of the inflection point was measured in practice and the visibility was forecasted.

In addition, numerous studies contributed to the image contrast models which are based on contrast threshold and gradient [13,14,15,16,17]. Boussard, et al. [13] focused on the study of low visibility condition. The depth map of vehicle environments was obtained with onboard cameras, and the contrast threshold value (5%) was used for visibility estimations. Hermansson and Edstam [14] raised a contrast calibration method that changed the weather background of outdoor images and was capable of inspiring the visibility estimation. The weather parameters incorporated atmospheric conditions, illumination, visibility, etc. Hautière, et al. [15,16] proposed a generic method for visibility estimation based on stereo vision, and the fog image was initially collected by the camera onboard. According to the atmospheric visibility definition given by CIE, the contrast threshold value (5%) was taken advantage of, and the real-time disparity contrast was combined. Graves and Newsam [17] put forward a prediction model for visibility estimation in view of the image contrast. Regression trees, multivariate linear regression, and a semi-supervised learning framework were used for the learning of the regression model. Besides, a set of images were utilized there.

Moreover, road signs, such as road lane line, pavement, traffic signs, etc., are frequently used to estimate the atmospheric visibility [18,19,20,21,22,23]. Based on [13], Bronte, et al. [18] proposed a real-time fog estimation system using onboard b&w camera. Three unlike levels—“sunny or cloudy with low fog”, “cloudy with medium fog”, and “cloudy with high fog”—can be estimated. Boussard, et al. [19] estimated the visibility distance in view of the structure from motion. The scene images were filmed by an onboard camera primarily, and the information of vehicle motion was extracted. Based on this, a spatial partial structure was established to evaluate the visibility distance. Lenor, et al. [20] estimated atmospheric visibility based on object tracks in the surveillance images, and he obtained the visibility and established the likelihood cost function for computing extinction based on the conventional Koschmieder’s formula. Belaroussi and Gruyer [21] estimated visibility with the knowledge of road signs in the digital map. The features of road signs were extracted, and the priori information implanted on the infrastructure was utilized, all of which were integrated together to estimate the fog visibility. There were relationships between the defog algorithm and the fog visibility estimation method, and the defog algorithm gave inspiration to foggy and hazy visibility estimation. He, et al. [22] proposed a simple but effective image prior-dark channel prior algorithm to remove haze from a single input image. Based on that, Song, et al. [23] presented a real-time visibility estimated method based on dark channel prior and lane detections. The variable box search (VBS) algorithm was raised for lane detections. To compute the extinction coefficient, two endpoints of one traffic lane were extracted.

Furthermore, the regression models of visibility estimation are presented in recent years. Some features are extracted from the foggy images and some relationship functions are then constructed [24,25]. Babari, et al. [24] come up with a visibility estimation means based on the gradient magnitude and Sobel gradient weighted. The fog video gathered from roadside cameras and the non-linear regression were used for calibration. Varjo and Hannuksela [3] assessed the fog visibility based on feature vectors and the high dynamic range imaging. Therefore, the quality of the night image can be enhanced and applied to the visibility estimation. Kim [25] presented a method with relevant knowledge of the chromatic analysis and a nonlinear function. A correlation between visibility and the vertical coordinate position of the visual images was established, and the visual range can thus be estimated.

The drawbacks of the vision based visibility methods above have been summarized in this paper (as shown in Section 1). To overcome the drawbacks, the total bounded variation (TBV) is introduced to design a new approach for atmospheric visibility estimation. The texture of image can be characterized by TBV and various practical applications in image processing were studied. Rudin, et al. [26] proposed a constrained optimization type of numerical noise removal algorithm based on TBV, and the noise statistics were employed to minimize the TBV of images. Rudin and Osher [27] made a research on the image restoration based on TBV and free local constraints of images. Chambolle and Lions [28] proposed an image recovery algorithm based on TBV minimization. Osher, et al. [29] put forward an iterative regularization means based on TBV, and the image was reestablished. Other image restoration and de-blurred algorithms in view of TBV were demonstrated in [30,31,32,33,34,35]. Cheng, et al. [36] proposed an image distortion metric based on TBV, and a complete mathematical derivation was then given. The result in [36] was that the bigger the TBV is, the clearer the image is, and vice versa. Based on the previous research above (vision based methods and TBV), the TBV approach will be introduced in details.

## 3. Research Methods

### 3.1. Visibility Definition and Application

Visibility reflects atmosphere transparency, which is closely related to floating fine and ultrafine particles in the atmosphere. Parallel light is scattered by floating particles involving water vapor coagulation and dry matter, and atmospheric visibility is varied as a consequence. In addition, visibility is linked to the observer’s visual ability and understanding. In addition, it is affected by some other factors like illumination and background. Hence, visibility estimation is a complex physical and psychological process. Based on the definition of International Commission on Illumination (CIE) [37], atmospheric visibility is the longest distance at which a black object with suitable dimensions can be recognized during daytime.

When visibility is short of 200 m [38], the driving speed should be lower than 60 km/h and the safe distance should be further than 100 m. When visibility is less than 100 m, driving speed should be lower than 40 km/h and the safety distance should exceed 50 m. When the visibility is no more than 50 m, the expressway ought to be closed and all vehicles should leave the expressway from the nearest exit. The speed should be lower than 20 km/h, and the danger alarm flash of vehicles should be turned on. In reality, most traffic accidents resulting from fog and haze occur on the expressway with foggy and hazy visibility of less than 200 m [39]. These facts motivate the study on the low visibility estimation in this paper.

**Figure 1:**
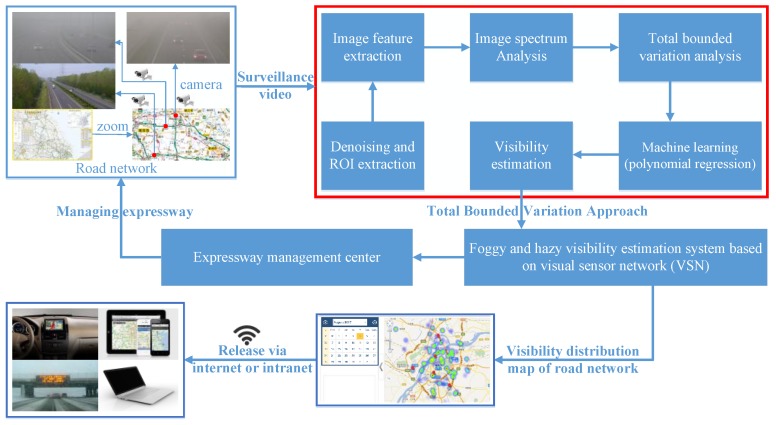
Practical application of total bounded variation approach.

At present, there is a set of high definition (HD) surveillance cameras every 5–10 km in China’s expressways. For those special sections with high traffic accident rates, the density of surveillance camera is one group/km, and the application of the visibility estimation algorithm is shown in Figure 1. The TBV approach presented in this paper is a sub-model of the “foggy and hazy visibility estimation system based on the visual sensor network”. The surveillance video of the road network is processed by the TBV approach, and the road network visibility map can be generated. With the estimated visibility information, the traffic flow can be controlled by expressway administrators. The foggy and hazy visibility map of the road network can be released through many ways, such as cell phones. Then, drivers can avoid dangerous sections, and the number of traffic accidents will be largely reduced.

### 3.2. Pseudo-Blurred Image

It is assumed that the occurrence of fog and haze is a linear and process. Suppose fog and haze are additive noise, and foggy and hazy images are superposition of fog (haze) and sunny dayimages in the same scene, fog and haze images thus are processed as blurred images (also named pseudo-blurred images in this paper). Assume that function *f*(*x*, *y*) denotes a sunny day image and the texture of *f*(*x*, *y*) is blurred by fog and haze; the pseudo-blurred image *g*(*x*, *y*), hence, can be obtained.

(1) g(x,y)=h(x,y)∗f(x,y)+n(x,y)

where *h*(*x*, *y*) is the spatial representation of the degradation function, and it is the blur filter for blurring image *f*(*x*, *y*). The symbol “*” indicates convolution, and *n*(*x*, *y*) is noise. Concerning simplicity, the noise item in Formula (1) is ignored, and then we can get the formula below

(2) $$g(x,y)=\int_{-\infty }^{\infty }\int_{-\infty }^{\infty }f(q,p)h(x-q,y-p)dqdp$$

where *h*(*x*, *y*) meets the constraints, that is

(3) $$\int_{-\infty }^{\infty }\int_{-\infty }^{\infty }h\left(x,y\right)dxdy=1,h\left(x,y\right)\geq 0$$

Formula (3) ensures that the blurred image *g*(*x*, *y*) is fuzzier than the sunny day image *f*(*x*, *y*). The heavier the fog and haze are, the deeper the degree of degradation of *f*(*x*, *y*) is and the lower the visibility is, and vice versa. For the foggy and hazy image with visibility of less than 300 m, the degradation is severe, and the image texture is blurry. Based on this feature, the foggy and hazy image can be filtered out by image spectrum.

### 3.3. Foggy and Hazy Image Spectrum

High and low frequency coefficients of image discrete cosine transform (DCT), the simplification of Fourier transform, are used to filter out low visibility images. The image is transformed from the spatial domain to the frequency domain, and the image frequency (spatial frequency) indicates the situation where the image pixel gray value changes in the spatial domain. Suppose the resolution of image *f*(*x*, *y*) is *n × n*, then the image spectral coefficient of *f*(*x*, *y*) can be obtained by DCT. It is an *n × n* spectral matrix. The upper left corner of the spectral matrix is a low frequency component indicating the smooth area of the image, and the gray value variety is small. The lower right corner is high frequency component which indicates large and fast gray value variety. *F*(0, 0) denotes the direct current (DC) component, and *F*(*u*, *v*) denotes the alternating current (AC) component. They are shown as Formulae (4) and (5).

(4) $$F\left(0,0\right)=\frac{1}{n}\sum_{x=0}^{n-1}\sum_{y=0}^{n-1}f\left(x,y\right)$$

(5) $$\begin{array}{l} F\left(u,v\right)=\frac{2}{n}\overset{n-1}{\underset{x=0}{\sum }}\overset{n-1}{\underset{y=0}{\sum }}f\left(x,y\right)\cdot \cos\left[\frac{\pi }{2n}\left(2x+1\right)u\right]\cos\left[\frac{\pi }{2n}\left(2y+1\right)v\right]\\ u,v=1,2,\cdots ,n-1 \end{array}$$

As mentioned above, foggy and hazy images are processed as noise blurred images, and the sunny day images are processed as clear images. Suppose that the foggy and hazy image is the result of convolution between sunny day image and foggy and hazy noise, the image spectral coefficient, based on the hypothesis can be calculated. The background differences in disparate road surveillance points are vast, resulting in wide differences in image spectrum. To overcome the effect of different road points, lighting and other factors, we use the relative ratio:

(6) $$\begin{array}{l} F_{r}\left(u,v\right)=\frac{F_{l}\left(u,v\right)}{F_{h}\left(u,v\right)}\times 100\%\\ u,v=1,2,\cdots ,n-1 \end{array}$$

where *F_r_*(*u*, *v*) denotes the DCT coefficient ratio and *F_l_*(*u*, *v*) denotes the DCT coefficient of foggy and hazy images with low visibility. *F_h_*(*u*, *v*) indicates the DCT coefficient of sunny day images with high visibility.

It should be emphasized that the pseudo-blurred images caused by fog and haze differ from the real blurred images caused by white noise, salt and pepper noise, or other noises. Firstly, foggy and hazy images are still clear images with HD, which are only assumed to be blurred images, and thus it is referred to as pseudo-blurred image in this paper. Secondly, fog and haze are continuous, and differences between local boundaries of images are smaller. The HF coefficient ratio of pseudo-blurred images fluctuates in a small range. For instance, when visibility exceeds 200 m, the HF coefficient ratio will steadily increase; when visibility is no more than 300 m, it will be less than a constant. Additionally, the smaller the visibility is, the larger the LF coefficient ratio is. The reason lies in that the texture of low visibility images is smooth, and the differences between image pixel gray values are limited. Based on this spectral feature, the foggy and hazy image (visibility is less than 300 m) can be classified.

### 3.4. Total Bounded Variation

After low visibility images are sorted out, the TBV method is applied for extracting foggy and hazy image features and characterizing the distinctions of image local boundaries, and the visibility, therefore, can be estimated.

Let *f*(*x*, *y*) denote the sunny day image which is processed as the clear image in this paper, and the varying rate of function *f*(*x*, *y*) in *x* and *y* directions can be calculated. Then, its absolute values and square of summation are computed, and the TBV of *f*(*x*, *y*) obtained is listed below:

(7) $$TBV_{f}=\int_{-\infty }^{\infty }\int_{-\infty }^{\infty }{\left[\left|\frac{\partial f(x,y)}{\partial x}\right|+\left|\frac{\partial f(x,y)}{\partial y}\right|\right]}^{2}dxdy$$

where the sunny day image is collected from the same road point with foggy and hazy images and the sunny day visibility surpasses 1 km. The start time of image collection is 12:00 and the duration is 50 min. Based on Formula (7), TBV of foggy and hazy image *g*(*x*, *y*) is

(8) $$TBV_{g}=\int_{-\infty }^{\infty }\int_{-\infty }^{\infty }{\left[\left|\frac{\partial g(x,y)}{\partial x}\right|+\left|\frac{\partial g(x,y)}{\partial y}\right|\right]}^{2}dxdy$$

For digital images, TBV of blur images is invariably less than TBV of clear image [36].

(9) $$\int_{-\infty }^{\infty }\int_{-\infty }^{\infty }{\left[\left|\frac{\partial g(x,y)}{\partial x}\right|+\left|\frac{\partial g(x,y)}{\partial y}\right|\right]}^{2}dxdy\leq {\int_{-\infty }^{\infty }\int_{-\infty }^{\infty }\left[\left|\frac{\partial f(x,y)}{\partial x}\right|+\left|\frac{\partial f(x,y)}{\partial y}\right|\right]}^{2}dxdy$$

**Figure 2:**
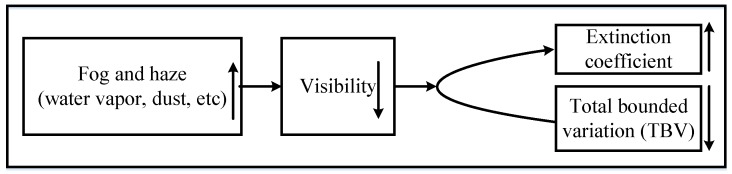
Relationship of atmospheric visibility, extinction coefficient and total bounded variation (TBV).

Inequality Formula (9) demonstrates that TBV of sunny day images is more than that of foggy and hazy images. At the same road point, the more the atmospheric visibility is, the larger the TBV is. The backgrounds of expressway surveillance points are relatively fixed and the TBV trend tends to converge to a constant number when visibility outnumbers 500 m. Therefore, the TBV of surveillance image in low visibility scene is proportional to the corresponding atmospheric visibility, which is shown in Figure 2.

Local boundary differences of images can be distinguished by TBV and the varying trend of TBV is identical with the trend of atmospheric visibility. Consequently, the nonlinear relationship function between TBV and real visibility is established based on piecewise stationary time series analysis. The function is listed as follows

(10) $$Vis_{n}=a_{n}{\mathrm{TBV}}_{n}^{2}+b_{n}{\mathrm{TBV}}_{n}^{1}+c_{n}{\mathrm{TBV}}_{n}^{0}$$

where *n* denotes visibility intervals, and coefficients *a_n_*, *b_n_* and *c_n_* are unlike in different visibility intervals. Based on machine learning, big data can be used for training, and *a_n_*, *b_n_* and *c_n_*, therefore, are obtained. In application, TBV is calculated by Formulae (7) and (8), and the atmospheric visibility can be obtained via Formula (10). Moreover, the original TBV value is large. To overcome the difference of road points background, the relative ratio of TBV as follows is used in this paper.

(11) $${\mathrm{TBV}}_{r}=\frac{{\mathrm{TBV}}_{l}}{{\mathrm{TBV}}_{h}}\times 100\%$$

where TBV*_l_* denotes the TBV of foggy and hazy images, and TBV*_h_* denotes the TBV of sunny day images. To compute the relative ratio, the sunny day images required in Formulae (6) and (11) are collected from the same road point at 12:00 and the duration is 50 min. The singular values (*F_h_*(*u*, *v*) or TBV*_h_*) are removed and the average value is computed. Detailed steps of the TBV approach presented are shown in Algorithm 1. To evaluate the performance of the TBV approach, we use relative error in this paper.

(12) $$error=\frac{Vis^{\prime }-Vis}{Vis}\times 100\%$$

where *Vis′* is the visibility estimated by the TBV approach and *Vis* is the real atmospheric visibility.

**Algorithm 1:** Total bounded variation approach to low visibility estimation**Input:** Surveillance video, 990 min × 60 s/min × 30 frame/s = 1,782,000 frames**Output:** S-TBV model, visibility *Vis***Initialization:** Sampling interval time**Step:**1. Surveillance video preprocessing and sampling; 2. ROI extraction based on different road points;3. Search low visibility frame (less than 300 m);  (1) DCT processing for sunny day images captured from the same road point (50 min);  (2) Analyze the HF and LF coefficients of images processed in Step 3.1, and their median value is used;  (3) DCT processing for fog and haze surveillance images;  (4) Analyze image spectrum, e.g., DC component *F*(0, 0) and *F*(*n* − 1, *n* − 1), and calculate relevant values based on Formula (6) and Steps 3.1–3.4;  (5) Search the low visibility frame on the basis of *F_r_*(0, 0) and *F_r_*(*n* − 1, *n* − 1);  (6) **Notes:** if the visibility of fog and haze is less than 300 m, go to Step 4, or stop and output message, which is “more than 300 m”.4. Compute foggy and hazy visibility;   (1) Calculate the TBV value for sunny day images in the same road point (50 min);  (2) Use the median value to analyze the TBV above;   (3) Calculate the TBV value for foggy and hazy images using the relative TBV;  (4) Piecewise stationary function construction with machine learning (polynomial regression)   **(1)** **Training set:** the coefficients *a_n_*, *b_n_* and *c_n_* in Formula (10) are obtained by training.   **(2)** **Testing set:** video data of road points 2, and 4–6 are used as the testing set respectively.   **(3)** **Notes:** the training set and the testing set are independent. For example, data of road Points 2 are used as the testing set, and data of other road points (1, 3–6) are used as the training set.5. Optimize algorithm parameters.The greater the amount of the training set is, the better the training effect is. Therefore, all data of 6 road points (1,782,000 frames) are used for S-TBV model training, and then coefficients *a_n_*, *b_n_* and *c_n_* in Formula (10) are obtained.

## 4. Results

**Figure 3:**
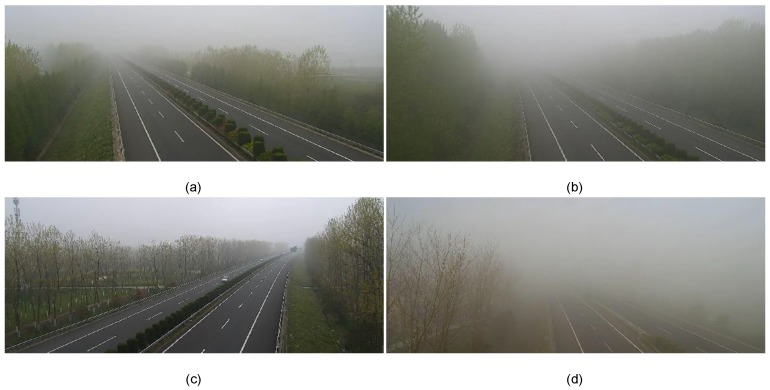
Foggy and hazy images (Sample images of road points 2–4, and 6 are shown in (**a**–**d**), respectively).

To validate the TBV approach presented in this paper, we analyze the foggy and hazy surveillance videos of expressways. The frames shown in Figure 3 are collected from Tongqi expressway (China) operated by the Intelligent Transport System (ITS) which works 24 h per day, in Jiangsu province of China.

In expressway sections where fog and haze happen frequently, a good deal of foggy and hazy videos are gathered with gradual variation. As shown in Table 1, the videos for six road points are all collected in the early morning. During the collection process, fog and haze disappear gradually until atmospheric visibility reaches 300 m. The HD video used in this paper is 990 min, and it has 990 min × 60 s/min × 30 frames/s = 1,798,200 frames.

Since the ITS system works 24 h per day, the sunny day videos needed by TBV approach can be collected easily. The parameters required in Formulae (6) and (11) can also be obtained, which are *F_h_*(0, 0), *F_h_*(*n* − 1, *n* − 1) and TBV*_h_*. The video is captured from 12:00, the duration is 50 min when the road point visibility is over 1000 m. In view of the data collection above, the spectral coefficients and TBVs of sunny day images are calculated. The average is computed after removing singular values and the results are used in Formulae (6) and (11).

The software platform for validating the TBV approach is Matlab 2017a, and the hardware incorporates CPU i7-5500U, 2.4 GHz, 16 GB RAM and double graphics cards. One graphics card is NVIDIA Geforce 940M and display RAM is 1 GB. From the application standpoint, the visibility estimation result will be used by the car drivers or the staffs of expressway control center. Therefore, the perception of human eye is significantly important. Based on the visibility definition of CIE, 36 subjects are invited for subjective assessment experiments of foggy and hazy visibility. Finally, the visibility estimated values in this paper are confirmed by the real visibility obtained from actual observations.

**Figure 4:**
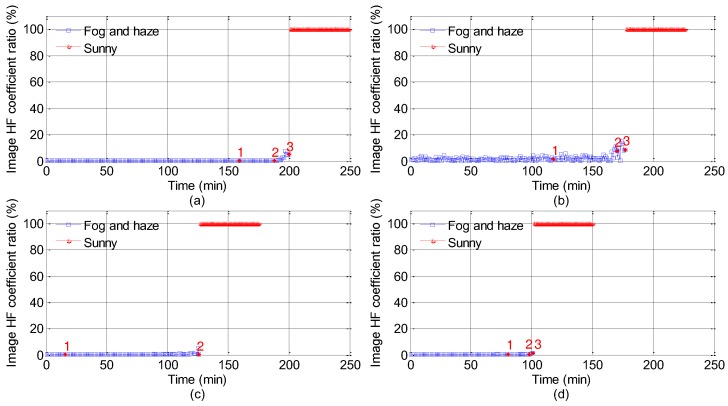
High frequency coefficient ratios ((*F_r_*(*n* − 1, *n* − 1) = *F_l_*(*n* − 1, *n* − 1)*/F_h_*(*n* − 1, *n* − 1). The red numbers 1, 2 and 3 indicate 100 m, 200 m and 300 m, respectively. The upper limit visibility in road points 5 is 200 m, and so there is no red number 3 in (**c**). The ratio values shown in (**a**–**d**) are corresponding to the Points 2, 4, 5 and 6, respectively.).

**Figure 5:**
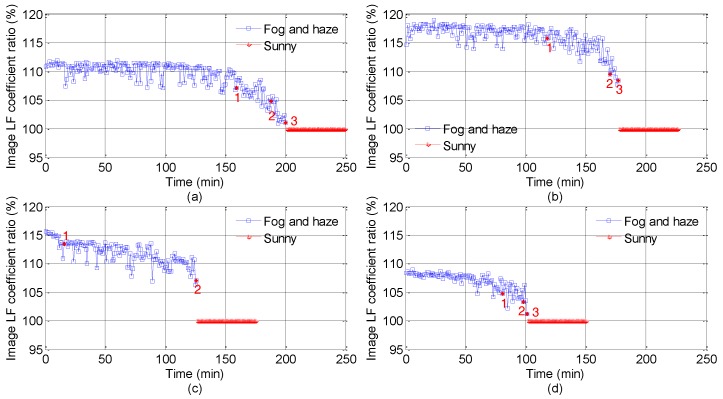
Low frequency coefficient ratios ((*F_r_*(0, 0) *= F_l_*(0, 0)*/F_h_*(0, 0). The red numbers 1, 2 and 3 indicate 100 m, 200 m and 300 m, respectively. The upper limit visibility in road points 5 is 200 m, and so there is no red number 3 in (**c**). The ratio values shown in (**a**–**d**) are corresponding to the Points 2, 4, 5 and 6, respectively.).

Figure 4 and Figure 5 show HF coefficient ratios and LF coefficient ratios, respectively. The blue data points are the spectral coefficient ratios of foggy and hazy images, and the red data points are the spectral coefficient ratios of sunny day images. It is not continuous between the blue data points and the read data points. Due to the huge background difference of road points in expressways, the disparity in the corresponding DCT coefficients are vast. To overcome the impact of relevant factors, such as road points background, light and camera angles, we use the relative value of the spectral coefficient computed in Formula (6). In Figure 4 and Figure 5, the red numbers (1, 2, and 3) reveal real visibility (100 m, 200 m, and 300 m), for the highest visibility in road points 5 is 200 m and there is no number 3 in Figure 4c and Figure 5c. For the sake of LF coefficient ratios of images, DC component *F*(0, 0) is adopted in this paper; for the sake of HF coefficient ratios of images, the AC component *F*(*n* − 1, *n* − 1) is applied. In the foggy and hazy dissipation process, fog and haze dissipate faster than before when atmospheric visibility varies from 200 m to 300 m. Hence, the corresponding surveillance frame number turns out to be smaller, and red sign number 2 draws near to red sign number 3 in Figure 4 and Figure 5. In general, there are two spectral features of foggy and hazy images in Figure 4 and Figure 5. Firstly, the whole *F_r_*(*n* − 1, *n* − 1) values of foggy and hazy images with low visibility (less than 300 m) are less than 20%, which is demonstrated in Figure 4. When the visibility goes up, *F_r_*(*n* − 1, *n* − 1) of corresponding foggy and hazy images will rise. Secondly, *F*(0, 0) values of foggy and hazy images with low visibility (less than 300 m) shown in Figure 5 are entirely between 100% and 120%. Based on the two spectral features, the foggy and hazy images with visibility of less than 300 m can be sorted out.

**Figure 6:**
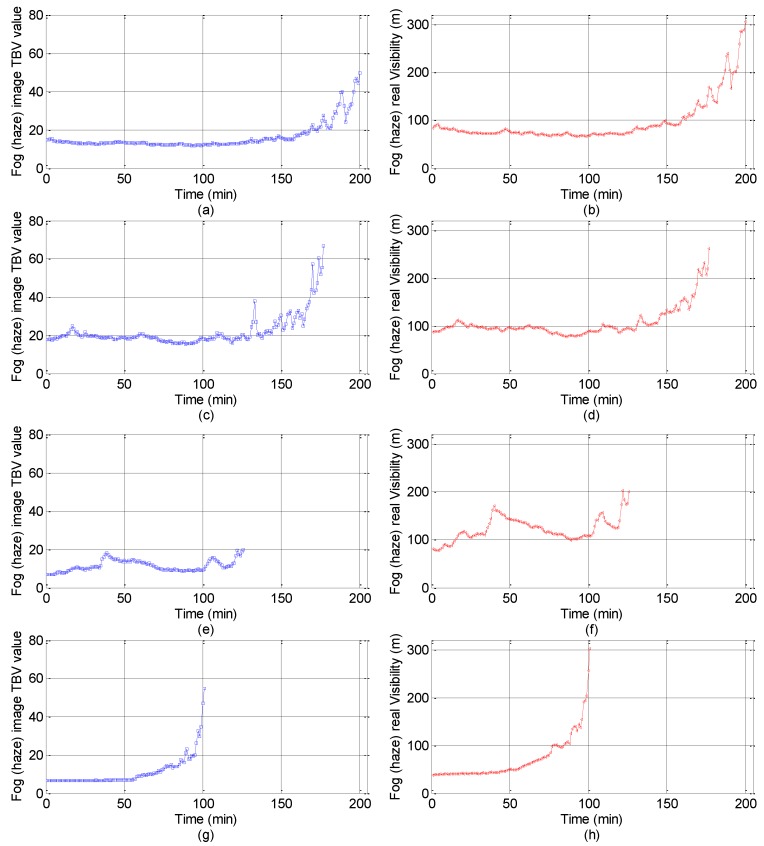
Varying trend comparison between the TBV ratio and the real visibility. (The data shown in (**a**,**c**,**e**,**g**) denote the TBV ratio values of Points 2, 4, 5, and 6, respectively. The data shown in (**b**,**d**,**f**,**h**) indicate the foggy and hazy visibility values of the Points 2, 4, 5, and 6, respectively.)

The varying trend comparison between TBV and real visibility can be seen in Figure 6. The left column of Figure 6 is the TBV varying trend of images during the foggy and hazy dissipation period, and the right column is the corresponding visibility. The data shown in Figure 6 correspond to road points 2, and 4–6 in Table 1. In reality, the TBV varying trend of images in road points 1, and 3 is identical with that of others. However, the data in road points 2, and 4–6, exclusively and obviously shown in Figure 6, are used as the testing set. Throughout the foggy and hazy disappearance process, the visibility varies from low to high levels, and there is fluctuation in a certain time interval. In general, the varying trend of TBV is completely consistent with the variation trend of foggy and hazy visibility.

**Figure 7:**
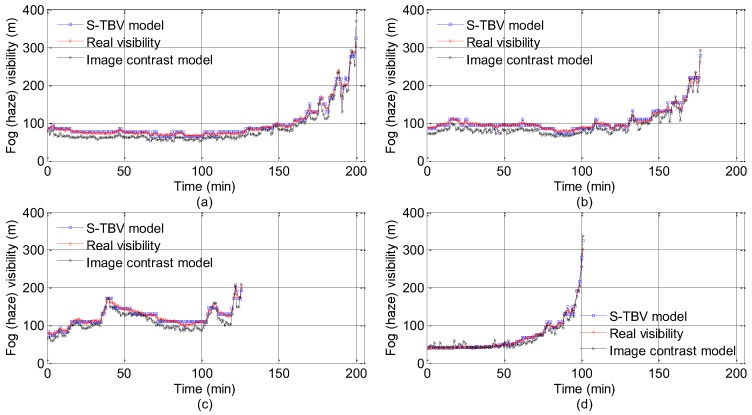
Visibility comparisons among the real values, the estimation results of the TBV approach and the results of the image contrast model (The visibility values shown in (**a**–**d**) are corresponding to the Points 2, 4, 5, and 6, respectively.).

**Figure 8:**
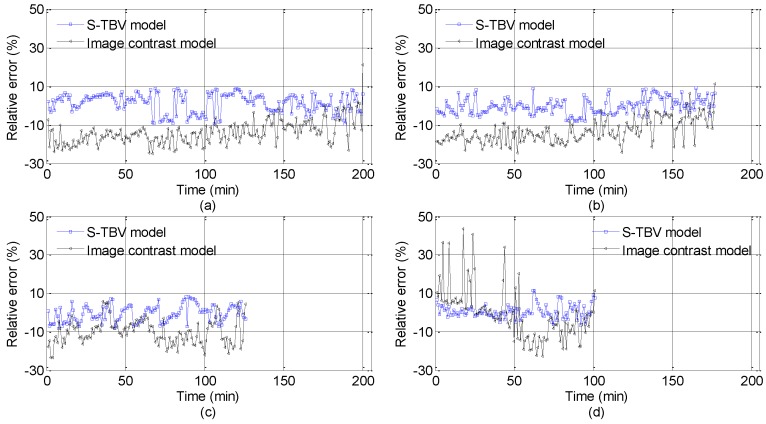
Relative errors comparison between the TBV approach and the image contrast model. (The image contrast model is based on [16] and the threshold 0.05 is used. The details about the relative error of the TBV approach are presented in Figure 9. The errors shown from in (**a**–**d**) are corresponding to the Points 2, 4, 5, and 6, respectively.)

**Figure 9:**
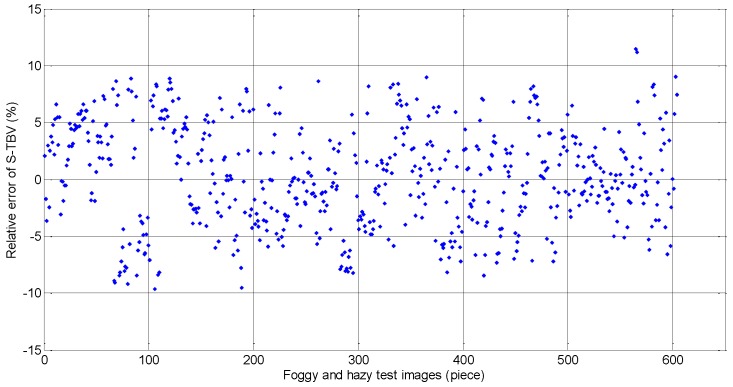
Relative errors of the TBV approach in detail. (The training set and the testing set are separated. A total of 604 foggy and hazy images are tested. There are only two relative errors which are between 11% and 12%. The other errors are less than 10%. Relative errors of 414 foggy and hazy images are less than 5%.)

In the process of TBV approach validation, the training set and the testing set are separated based on the machine learning theory. The TBV approach is tested by big data in the training set which is used for algorithm validation to get relevant parameters. The training and testing set information is shown in Table 2 and the test results are presented in Figure 7, Figure 8 and Figure 9. In Figure 7, the visibility estimated by the TBV approach is very close to real visibility, and it should be noted that the front visibility value estimated is similar to the back one in some short time intervals. The reason is that the piecewise stationary theory is applied in visibility. The estimation visibility of the previous frame is used as a reference for the visibility estimation of the next frame, and it is also, in reality, compliant with the characteristics of fog and haze. The estimated errors shown in Figure 8 and Figure 9 indicate the effectiveness of the idea above. In 604 measured foggy and hazy images, there are only two images whose relative errors are between 10% and 15%, and relative errors of other images are all less than 10%. There are 414 (68.54%) error data points with the percentage of less than 5%. According to [39], the upper limit of estimation errors is 10% if the atmospheric visibility is less than 2000 m, and the test results of the TBV approach presented in this paper obviously meet this requirement.

To further verify the effectiveness of the TBV approach, the image contrast model of visibility estimation [16] is used for performance comparison. The estimation results of the image contrast model are also shown in Figure 7 and Figure 8. Among 604 measured images, relative errors of 208 images (34.44%) are less than 10%, while 94 images have relative errors less than 5% (15.56%).

**Figure 10:**
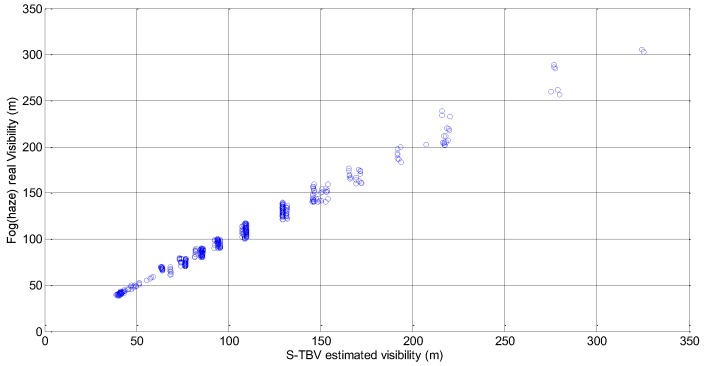
The scatter plot between the visibility estimated by the TBV approach and the real visibility.

Figure 10 is a scatter plot between real visibility and the visibility estimated by the S-TB algorithm. Figure 10 indicate the large amount of data points with visibility of less than 200 m, since the fog and haze dissipate slowly when the corresponding visibility is no more than 200 m. From the perspective of machine learning, the more the training data are, the better the S-TBV model is. As a result, all the foggy and hazy image data are used for S-TBV model training and the piecewise function coefficients are shown in Table 3.

## 5. Discussion

In all the collected data, the foggy and hazy images with visibility of less than 200 m can always be obtained because of the dissipation characteristics of fog and haze. When fog forms at midnight, the atmospheric visibility reaches its minimum. However, there is sometimes an “elephant trunk phenomenon” and visibility suddenly becomes better before reaching the minimum. While fog disappears gradually after sun rises, fog dissipation, is relatively slow and the characteristic of a piecewise stationary process is obvious when the atmospheric visibility is between 0 m and 200 m. As the fog with visibility of less than 200 m has long duration and is more dangerous, some special management rules for Chinese expressways are made in this situation [38].

The validation results show that TBV can be employed to characterize local boundary differences of foggy and hazy images. In the sunny day images, the shape of histogram distributions is bimodal. The TBV of the sunny day images is big because the difference among gray values of adjacent pixels is wide. When fog and haze occur, the histogram distribution shape of surveillance images tends to be unimodal or trapezoidal. To contrast, the corresponding TBV is low as the difference between gray values of adjacent pixels is smaller. In general, the better the visibility is, the higher the TBV is, and vice versa.

From the angle of image spectrum, the energy of fog and haze is chiefly concentrated in the low frequency region, and the HF coefficients are low with its ratios shown in Figure 4 between 0% and 20%. When heavy fog and haze occur, the variations between local boundaries of surveillance images are small, and the LF coefficients augment. As shown in Figure 5, the LF coefficients range between 100% and 120% when the visibility is less than 300 m. Based on the spectral features shown in Table 4, low visibility foggy and hazy images can be sorted out.

As mentioned above, the training set and the testing set are independent and the S-TBV model is validated. In Figure 9, the number of points with relative errors of less than 5% is 414, which accounts for 60% of the whole testing data. Only two data points have relative errors of more than 10%, i.e., 11.10% and 11.48%, respectively. The validated results meet and exceed the requirements shown in [39], where the estimated errors should be less than or equal to 10% when the visibility is less than 2000 m. In view of the validated data above, we can see clearly that TBV is closely linked to the features of fog and haze. A nonlinear relationship exists between TBV and real visibility, which can be analyzed by piecewise stationary theory. When visibility is less than 300 m, texture features of corresponding foggy and hazy images are apparent, and TBV is a well-designed parameter for characterizing such local differences among images. Furthermore, the piecewise stationary time series analysis theory is used for setting up the piecewise function between TBV and real visibility.

Some researchers may argue why the visibility values measured by the visibility meter were not used for performance comparison in this study. Reasons are summarized as follows: (1) the image contrast model has been used for performance comparison; and (2) perceived results by human eyes are more important than results based on objective measurements and predictions by different algorithms. Based on the visibility definition by CIE, 36 subjects were invited to participate in subjective assessments in this study.

This study aims to solve the Chinese practical problem. According to China meteorological industry standard [39], the performance of this study meets the practical application requirements (as shown in Section 4). Therefore, the validation performance is encouraging. However, it should be noted that there are still certain limitations. For example, the collection method for sunny day image can be improved. Furthermore, the big data in this paper are only collected from China. To improve the adaptability of the TBV approach, more data from different regions and countries are required to optimize the TBV approach.

## 6. Conclusions

Heavy traffic accidents usually result from fog and haze. When visibility is less than 200 m, the traffic accident rate will surge [39]. To alleviate this problem, a total bounded variation approach to estimate foggy and hazy visibility was studied, and it focuses on the expressway visibility of less than 300 m. The conclusions are listed as follows:

(1) The situation, expressway visibility of fog and haze less than 300 m, was focused on firstly. This strategy of overcoming the challenge of estimation accuracy is reasonable. The total bounded variation approach can be used to handle this situation and the verified results are encouraging. The relative errors of estimation are less than 10%, and 68.54% of the errors are less than 5%.
(2) Total bounded variation approach provides an effective framework for low visibility estimation on expressway. The fogy and hazy images can be processed as pseudo-blurred images. The texture features of pseudo-blurred images can be characterized by TBV, which is correlated to the trend of foggy and hazy visibility.
(3) There are wide differences between the spectral features of sunny day images and that of foggy and hazy images of less than 300 m. Besides, HF coefficient ratios of sunny day images fluctuate around 100%, and HF coefficient ratios of foggy and hazy images fluctuate from 0% to 20%. LF coefficient ratios of foggy and hazy images fluctuate from 100% to 120%, and LF coefficient ratios decrease gradually when the visibility increases steadily.
(4) Big dataset can help generate valid the S-TBV model. The dataset contains 1.78 million frames collected from expressway.
(5) Relative ratios are used in this paper, namely the spectral coefficient ratio (HF, LF) and the TBV ratio. Some influencing factors, such as road points background and lighting, can be eliminated. The feasibility of the S-TBV model will be improved.

The results of the TBV approach presented are satisfactory. Moreover, it would be very meaningful to have a comparison between the results of this paper and that of deep learning. Deep learning can help train an end-to-end mapping from video frames to visibility. Furthermore, the nighttime visibility analysis is a valuable research topic, and the impacts of headlights on low visibility estimation deserve deep consideration in this topic. These will be our future works.

## Figures and Tables

**Table 1:** Road points information.

Road Points No.	Chainage	District	Start and End Time	Duration	Maximum Visibility	Date
1	K113 + 000	Dasheng	06:30–09:22	172 min	306 m	14 April 2016
2	K148 + 150	Haimen	06:00–09:20	200 min	306 m	14 April 2016
3	K159 + 950	Haimen	06:00–09:34	214 min	315 m	14 April 2016
4	K106 + 980	Dasheng	06:00–08:57	177 min	262 m	14 April 2016
5	K159 + 950	Haimen	06:00–08:06	126 min	200 m	13 April 2016
6	K208 + 027	Chenqiao	06:00–07:41	101 min	303 m	15 March 2016

**Table 2:** Training and testing sets.

Testing Set		Training Set	
Road Points No.	Duration	Road Points No.	Duration
2	200 min	1, 3, 4, 5, 6	790 min
4	177 min	1, 2, 3, 5, 6	813 min
5	126 min	1, 2, 3, 4, 6	864 min
6	101 min	1, 2, 3, 4, 5	889 min

**Table 3:** Piecewise function coefficients for the S-TBV Model.

*n*	*a_n_* (Power = 2)	*b_n_* (Power = 1)	*c_n_* (Power = 0)	Intervals
1	14.06	−169.68	548.13	[0, 50)
2	2.54	−37.20	187.41	[50, 60)
3	−6.52 × 10^−4^	−5.31 × 10^−2^	68.62	[60, 70)
4	−6.71 × 10^−4^	1.06 × 10^−1^	73.01	[70, 80)
5	−1.17 × 10^−2^	5.93 × 10^−1^	79.23	[80, 90)
6	−9.11 × 10^−3^	4.29 × 10^−1^	90.52	[90, 100)
7	7.62 × 10^−3^	−4.28 × 10^−1^	112.77	[100, 120)
8	−4.53 × 10^−4^	5.31 × 10^−3^	128.93	[120, 140)
9	−3.27 × 10^−3^	4.52 × 10^−1^	140.98	[140, 160)
10	3.22 × 10^−3^	−2.16 × 10^−1^	170.96	[160, 180)
11	−2.45 × 10^−3^	1.34 × 10^−1^	189.99	[180, 200)
12	5.07 × 10^−3^	−3.27 × 10^−1^	220.76	[200, 250)
13	−1.11 × 10^−2^	1.33	240.49	[250, 300]

**Table 4:** Spectral features of foggy and hazy images.

DCT Coefficient Ratios	Foggy and Hazy Image Spectrum	Overall Trend
High frequency (HF)	Overall between 0% and 20%.	When the foggy and hazy visibility is improved, the HF coefficient ratios increase.
	Fluctuating around a small number when the visibility is less than 200 m.	
	Increasing gradually when the visibility is between 200 m and 300 m.	
Low frequency (LF)	Between 100% and 120%.	The higher the foggy and hazy visibility is, the smaller the LF coefficients are.
